# An Improved Physical-Stochastic Model for Simulating Electrical Tree Propagation in Solid Polymeric Dielectrics

**DOI:** 10.3390/polym12081768

**Published:** 2020-08-07

**Authors:** Johnatan M. Rodríguez-Serna, Ricardo Albarracín-Sánchez, Isabel Carrillo

**Affiliations:** 1 Departamento de Ingeniería Eléctrica, Electrónica, Automática y Física Aplicada, Escuela Técnica Superior de Ingeniería y Diseño Industrial (ETSIDI), Universidad Politécnica de Madrid (UPM), Ronda de Valencia 3, 28012 Madrid, Spain; johnatan.rodriguez.serna@alumnos.upm.es or; 2 Departamento de Ingeniería Eléctrica, Facultad de Ingeniería, Universidad de Antioquia (UdeA), Calle 67 No. 53–108, Medellín 050010, Colombia; 3 Departamento de Ingeniería Mecánica, Química y Diseño Industrial, Escuela Técnica Superior de Ingeniería y Diseño Industrial (ETSIDI), Universidad Politécnica de Madrid (UPM), Ronda de Valencia 3, 28012 Madrid, Spain; isabel.carrillo@upm.es

**Keywords:** electrical tree propagation, epoxy resin, physical-stochastic models, insulation ageing, dielectric breakdown

## Abstract

The dielectric breakdown of solid polymeric materials is due to the inception and propagation of electrical trees inside them. The remaining useful life of the solid dielectrics could be determined using propagation simulations correlated with non-intrusive measurements such as partial discharges (PD). This paper presents a brief review of the different models for simulating electrical tree propagation in solid dielectrics. A novel improved physical-stochastic model is proposed, which allows quantitatively and qualitatively analyzing the electrical tree propagation process in polymeric dielectrics. Simulation results exhibit good agreement with measurements presented in the literature. It is concluded that the model allows adequately predicting the tree propagation behavior and additional experimental analyses are required in order to improve the model accuracy.

## 1. Introduction

Insulation systems are designed in such a way that the electric fields applied to the materials are lower than the inception values for breakdown mechanisms. However, due to unforeseen manufacturing problems or operating conditions, metal contaminants, mechanical defects, conducting projections or air-filled cavities may appear, causing excessive local electrical field stresses within small regions of the solid dielectric [1,2]. These defects can lead to a complete breakdown of the dielectric through the formation of tubular structures made up of gaseous channels of microscopic diameter, known as electrical trees, that spread across the material by a mechanism controlled, mainly, by partial discharges (PD) in the gaseous channels [3,4].

The dielectric breakdown in solid insulation, such as polymers and synthetic resins, used in electrical equipment insulation systems such as cables, circuit breakers and bushings, is due to the appearance of electrical trees [5,6]. Therefore, it is necessary to study the behavior of electrical trees in solid dielectrics in order to understand the phenomenon and predict the useful life times, or in other words, the time to breakdown [7].

PD activity directly influences the aging of insulation, with physical–chemical degradation in the area of its occurrence. This electrical insulation aging is due to a combined effect of thermal, electrical, ambient and mechanical stresses [8]. The electrical aging involves a permanent change in the polymer’s chemical and structural features, which could accelerate the physical and chemical aging of the polymers that weakens their ability to sustain electric stress [9].

The continuous PD activity may modify the dielectric properties of solid dielectric materials making them less effective as electrical insulators. In spite of different chemical composition and morphology among the insulating polymeric materials, they shared a similar behavior to the breakdown previously described by [10].

To increase the properties of these materials in terms of mechanical and electrical erosion reduction, mechanical strength enhancement, electrical breakdown/endurance behavior, lower dielectric permittivity, less space charge mitigation and less electrical tree formation, new materials such as polymer nanocomposite have been developed [11,12,13]. Further, different nanofillers such as silicon dioxide (SiO_2_), aluminum oxide (Al_2_O_3_), zinc oxide (ZnO), magnesium oxide nanofiller (MgO), titanium oxide nanofiller, and montmorillonite nanofiller have been used [14]. However, some problems such us a decreased breakdown strength have been detected when nanofillers are aggregated [14] that has questioned their reliability and applications. To avoid agglomeration, nanofillers are modified using chemical coupling agent, which improves the dispersion of the nanofiller considerably [15]. Different surface modification strategies for the amelioration of nanofiller dispersion in the polymer matrix have been intensively attempted by linking the small organic molecules or long polymeric chains on the surface of nanofillers [15,16]. Another important factor is the concentration of fillers due to the trade-off between the difficulty in film processing and the enhancement of dielectric properties [17].

Degradation of polymers is a process driven by physical, chemical, thermal and mechanical factors [4]. In the case of electrical trees in solid dielectrics, from a physical point of view, its propagation is due to the breakdown of polymer chains, which is caused by a large number of interactions between molecules and electrons with high kinetic energy [18]. The electrical trees initially propagate quickly and then stop. This behavior has been related to changes in pressure and the PD rate [9,19,20]. The spatial charge around the tree structure limits the magnitude of the local electric field and acts as a negative feedback term in the dynamics of the tree propagation process and its relation to the parameters that cause them, such as the applied voltage, dictate the propagation regime [21]. If the negative feedback term dominates, the tree propagation process stops; and if the forcing term dominates, a runaway uncontrolled growth process occurs. On the other hand, if there is a balance between forcing and negative feedback terms, the tree propagation slows down. This explains the shape of the characteristic curve of propagation length versus time [22].

This paper is organized as follows: first, the materials and methods are presented in Section 2. Second, a brief review of the current models for electrical-tree propagation in solid dielectrics is depicted in Section 3. Third, an improved physical-stochastic model for simulating tree propagation in polymeric materials is presented in Section 4. Fourth, a case of study and the simulation results are discussed and compared with real measurements in Section 5. Finally, some conclusions are depicted in Section 6.

## 2. Materials and Methods

The performance of the improved model proposed here will be analyzed by comparing simulation results with experimental measurements reported in [22], where the authors experimentally studied tree propagation in solid dielectrics under various applied voltages. The authors used CT200 and CY1311 epoxy resins manufactured by Ciba-Geigy, Basel, Switzerland. CT200 is a diglycidil ether of bisphenol A epoxy resin and CY1311 is a flexibilized bisphenol A epoxy resin, and both are used in insulators, bushings and circuit breakers [23]. Authors in [22] implemented a pin–plane arrangement with voltages between 6 and 16 kV RMS, AC, 50 Hz, the pin tip radius was 2 μm and the pin tip–plane distance was 2.25 mm.

For the simulations presented in this work, the electric potential and electric field strength distributions for each propagation step are calculated according to the Coulomb–Gauss law:

(1) ∇⋅D=ρ, 

where D=εE (C·m^−2^), *ρ*(C·m^−3^) is the free charge density and *ε*(F·m^−1^) is the permittivity of media. Equation (1) is solved using the finite differences and relaxation methods [24], with the potentials at the pin, plane and tree segments as boundary conditions. Because the conductivity in the tree structure is higher than that in the dielectric bulk, charges are injected in the dielectric between the electrodes during the tree propagation, which modify the potential distribution. This phenomenon is modelled using the relative conductivity approach presented in [25]. The electric potential at each lattice site in the dielectric, previously discretized using a 2D uniform squared lattice, is calculated as:

(2) $$U=U_{int}+U_{ext},$$

where $U_{int}$ (V) is the electric potential calculated from Equation (1) when the potential at the tree structure and pin are equal to $U_{int}^{0}$ (V). Similarly, $U_{ext}$ (V) is the electric potential calculated from Equation (1) when the potential at the tree structure and pin are equal to $U_{ext}^{0}$ (V) and ρ=0 (C·m^−3^) and $\sigma_{ch}=U_{int}^{0}/U_{ext}^{0}$. $U_{ext}^{0}$ (V) corresponds to the electric potential applied to the pin tip $U_{0}$ (V). More details are given in the results and discussion section.

## 3. Brief Review of Current Models for Simulating the Propagation of Electrical Trees in Solid Dielectrics

For the purposes of this paper, it is considered that the time required for the first channel growth, *t* < 200 s, is very low compared to its propagation time, *t* > 10 × 10^3^ s [26]. The models for simulating tree propagation through solid dielectrics can be classified as follows:

1. Stochastic,
2. Physical,
3. Empirical,
4. Deterministic,
5. Multiphysical, and
6. Cellular Automata.

### 3.1. Stochastic Models

Stochastic models are based on the model proposed by Niemeyer, Pietronero and Wiesmann, also known as the NPW model [27]. In this model, it is considered that the tree structure grows discretely and the propagation probability in a given direction depends on the magnitude of the local electric field strength raised to a power *η*, that allows controlling the fractal dimension of the tree structure. This model has been used for various studies such as statistical and probabilistic analyses [28,29,30], the evaluation of the effect of dielectric barriers [5,31] or the space charge on tree propagation [3], and tree propagation in inhomogeneous media or including space charge [25]. The main disadvantages of this model are that the power *η* does not have a physical relationship with the tree propagation phenomenon and the time is not included as a physical variable in the model.

### 3.2. Physical Models

The physical or avalanche model (DAM) is based on the electric aging and dielectric breakdown model proposed by Bahder et al. [32]. In this model, it is assumed that the PD in a cavity or tree channel induce avalanches within the insulation at the points where the PD end and the number of ionizations in an avalanche is considered to be proportional to the damage it produces [33]. The repetition in time of this process causes the damage to accumulate until it reaches a critical level, when a new channel will form. The length of each new channel is assumed equal to 10 μm from Hozumi’s and Okamoto’s experimental observations [34].

For calculating the local electric field strength, a regular squared lattice is defined in the solid dielectric and a potential drop is assigned to each bond, which allows considering the effects of charge distribution at the end of the channel and that the entire structure of the tree is not necessarily discharged each time an avalanche is induced. In addition, stochastic variations of that voltage drop also allow modelling local fluctuations of the electric field resulting from the breakdown mechanism itself or from spatial non-homogeneities in the electrical properties of the insulation [21,35]. Simulation results using this model are consistent with Noto’s and Yoshimura’s experimental measurements [36], in addition, this model allows a quantitative description of the dependence between tree propagation and applied voltage [26].

### 3.3. Empirical Models

Empirical models use analytical expressions obtained through curve-fitting processes to experimental results. In [22], an empirical field-driven model was presented that allows calculating the length–time relationships during tree propagation. Using this model, it was concluded that there is a linear correlation between the fractal dimension and the breakdown time. This same model was used in [37] for studying the effect of the manufacturing age of the samples on the tree-propagation process in epoxy resins. It was found that there is a time window in which the resin is more prone to breakdown—60 to 200 h for the CT200 resin, in a pin–plane arrangement with 15 kV, 50 Hz, AC voltage, 2 mm electrode gap and 3 μm pin tip radius.

### 3.4. Deterministic Models

In [38], a physical-deterministic model for tree propagation was presented in which the damage to the material surrounding the tree is calculated using the dissipation of electrostatic energy due to PD in its gaseous channels. This model allows obtaining branched trees without the need to use a random variable, since fluctuations in the electric field strength are controlled by PD. The simulation results, propagation length–time curve and growth rate, as a function of voltage, are consistent with experimental measurements in non-conductive trees. Furthermore, it is shown that the branched nature of the tree structures occurs, mainly, due to instabilities in the electric field caused by the electric charge of the PD, rather than by the non-homogeneities in the material.

### 3.5. Multiphysical Models

Recently, multiphysical models for tree propagation have been proposed based on the model presented in [39]. The breakdown of solid dielectrics occurs due to the propagation of plasma channels in the solid. The propagation of plasma channels is considered to be due to electromechanical and electrothermal instabilities as well as ionization processes, in turn, produced by the dynamic behavior of electric fields, charge and energy within the channels and the dielectric material [40].

In [41], a physical-kinetic model was presented for the propagation of electrical trees in polymeric dielectrics, which allows evaluating the useful life of the insulation as a function of the applied voltage, the ambient temperature and the fractal dimension in a single equation. In this model, it is considered that the propagation occurs through microdefects around the tree, so the time interval between two consecutive tree length increments depends on the random distribution of the microdefects in the material. In [42], a variation to the previous kinetic model was presented to include the effect of residual compressive and tensile stresses on the tree propagation process. It was found that residual compressive stresses tend to contract the discharge channel, which tends to slow down the tree propagation, while tensile stresses tend to open the channels, which accelerates the tree propagation.

### 3.6. Cellular Automata Models

Cellular automata is a method that allows analyzing physical systems, which are discrete in time and space, using only local interactions [43]. This method has been used to simulate the propagation of electrical trees in solid dielectrics applying pin–plane arrangements and considering conditions of non-homogeneity in the permittivity of the material that may be a consequence of chemical deterioration processes of elementary volumes [44]. Further, it has been used to study the effect of space charge on tree propagation [45]. In [46], the tree propagation was studied in a polymeric material, polymethylmethacrylate, containing a conductive spherical particle, modelled as a region that has infinite permittivity, under applied DC voltage conditions. In [47], this model was used to study the influence of nanoparticles (NPs) on the propagation of electrical trees. Dielectric NPs are simulated as 100 × 100 nm rectangles, with a permittivity of twice the value corresponding to the base dielectric. The NPs are arranged in uniform configurations of up to 12 NPs in front of the pin tip electrode. It was demonstrated that the NPs, together with the space charge, act as a barrier that slows down the tree propagation.

## 4. A Novel Improved Physical-Stochastic Model for Simulating Tree Propagation in Polymeric Materials

From the analysis of the state of the art and the study of the tree propagation process in solid dielectrics, a new physical-stochastic model is proposed. The proposed model is based on the DAM model [33], but unlike in that, the time required to form a new channel in the new model is calculated from an analysis of the change on the potential energy in the dielectric.

When the electrical tree propagates along a specific path, depending on the local electric field strength magnitude, the time average electric potential energy around the new channel increases due to the increment in the electric field strength magnitude induced by the electric potential in the tree structure. In our model, it is considered that the increment in the potential energy is produced by the tree propagation and, according to the Poynting’s theorem [24], this increment must be directly proportional to the energy required for the new channel development. Therefore, the increment in the potential energy around the new channel will be equivalent to the kinetic energy transferred by the electrons after avalanches and can also be associated with the dielectric material vaporization energy [48,49].

On the other hand, the proposed model allows analyzing tree propagation in non-homogeneous media or cases where there is space charge, as shown in Equation (1).

In this model, as in the DAM model, it is assumed that avalanches occur during PD. The charges left by the PD at the interface between the solid dielectric and the gaseous channels intensify the electric field strength at the points of the solid dielectric beyond that interface and electron avalanches are induced within the propagation distance, which from experimental measurements is assumed as $L_{b}=10$ μm [34]. This is the length of each new channel that is added during the time step Δ*t* (s). Each electron is accelerated by the local electric field and acquires a kinetic energy that can be high enough to ionize the molecules in its path and thus generates more charge carriers. This process continues until the electrons cannot gain more kinetic energy for ionization and are thermally trapped. It is considered that due to the PD in the gas channels, there are enough available electrons for starting avalanches from the channels surface.

On the other hand, it is assumed that the electric field strength is constant during each avalanche. This can be justified taking into account the short duration of PD, <100 ns [50]. Furthermore, the media are considered to be linear and isotropic and only one channel is added at each time step. This is consistent with the discrete propagation behavior experimentally observed by other authors [51].

The channels are the result of polymeric chains, C–C bonds, scission due to consecutive avalanches caused by PD under AC voltage conditions. Electron avalanches involve the existence of inelastic collisions with the ion network in the polymeric material (polyester, polyethylene, and epoxy resins, among others), and therefore, the creation of a new channel implies, in turn, that the potential energy of the system changes [52].

If *λ* (m) is the mean free path in direction of the local electric field strength magnitude *E* (V·m^−1^), then the average energy gained by an electron in that same distance is ΔW=eEλ·(J). To cause impact ionization, this energy must be greater than or equal to the ionization energy of the molecule, *I* (eV). Assuming the Classius distribution [52] and if it is considered that said distribution will not be altered by the speed of the electrons in the field direction, all the electrons that acquire an energy greater than or equal to *I* (eV), will cause ionization. The number of ionizations per avalanche, $N_{A}$, can be calculated as [33]:

(3) $$N_{A}=\left[\exp\left(\alpha \left(E\right)L_{b}\right)-1\right],$$

where α(E) is the ionization coefficient, defined as the number of ionizations per electron per unit of path length and corresponds to:

(4) α(E)=(1/λ)exp[−I/(eλE)],

In solid materials, *λ* (m) and *I* (eV) are treated as adjustable parameters. In polymeric insulation, they are taken as 60 nm and 9.6 eV, respectively [53].

The energy transferred to the solid polymeric after an avalanche will be $\Delta W_{t}=N_{t}\Delta W$·(J). The number of ionizations in a period of time, *t* (s), of an AC voltage signal of frequency *f* (Hz), when there are $N_{b}$ avalanches per half cycle, can be calculated as [33]:

(5) $$N_{t}=2ftN_{b}\left[\exp\left(\alpha \left(E\right)L_{b}\right)-1\right],$$

The energy gained by the ion network in that time will be $\Delta W_{A}=N_{A}\Delta W$·(J). A channel will be created after a time, $t_{ch}$ (s), during which, from Equation (5), there will be the following number of ionizations:

(6) $$N_{c}=2ft_{ch}N_{b}\left[\exp\left(\alpha \left(E\right)L_{b}\right)-1\right],$$

$N_{c}$ is the critical number of ionizations for a new channel development. In polymeric materials, from measurements in polyester, $N_{c}=1\times 10^{13}$ [41]. The energy transferred to the ion network during this time will be $\Delta W_{c}=N_{c}\Delta W$·(J), and the time for a new channel development is:

(7) $$t_{ch}=\frac{\Delta W_{c}}{\Delta W}\frac{1}{2fN_{b}\left[\exp\left(\alpha \left(E\right)L_{b}\right)-1\right]},$$

where $\Delta W_{c}$ (J) is calculated as the change in the average potential energy around the new channel before and after its creation using the following equation:

(8) $$w_{c}=\frac{1}{4}\varepsilon E^{2},$$

where $w_{c}$·(J·m^−3^) is the local average potential energy density. With this model, it can be considered that the damage is accumulated in each region of space through the energy density stored there.

The creation of a channel at a specific point in the tree structure will be a stochastic process that will depend on the magnitude of the local electric field strength, as long as the conditions for ionization are met. From ΔW=eEλ·(J), a critical value of the electrical field strength for the tree propagation can be calculated as:

(9) $$E_{c}=I/e\lambda ,$$

This critical value can also be calculated from the definition of the ionization coefficient function, Equation (4), establishing a lower limit from which α(E)>0 and is approximately equal to 37 kV·mm^−1^ when I=18.5 eV [26]. The probability that a channel is added to the tree structure from a point will depend on the magnitude of the electric field strength there and is calculated as:

(10) $$p\left(E^{\eta }\right)=\{\begin{array}{c} E^{\eta }/\sum_{i}^{z}E_{i}^{\eta },E\geq E_{c}\\ 0,E<E_{c} \end{array},$$

where *η* has the same function as in the NPW model, and although it is clear that it does not have a physical relationship with the tree propagation process, it is included here taking into account that its value allows adequately controlling the fractal geometry of the tree structure [54]. Furthermore, experimental evidence suggests that electrical trees are deterministic processes that exhibit certain chaos in propagation [55] and it is considered that said behavior can be modelled with the value of *η* because this defines the oscillations of the stochastic model to a certain extent. The summation in Equation (10) includes all the probable bonds around the current tree structure.

**Figure 1:**
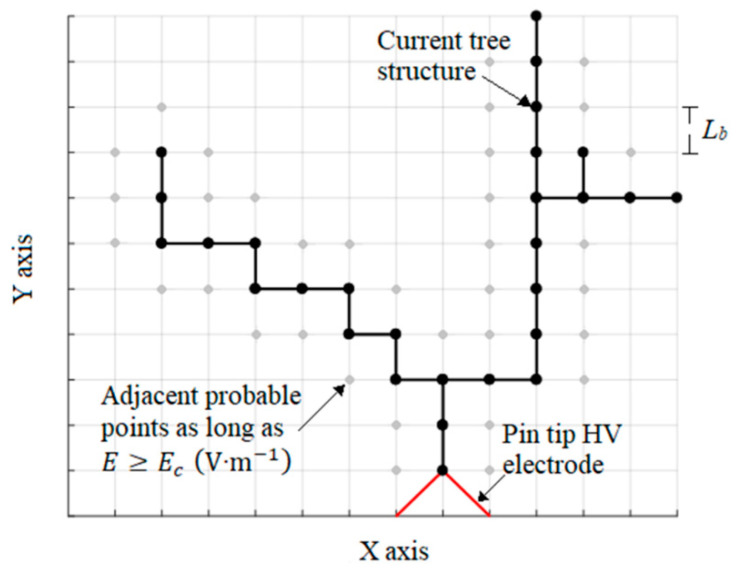
Tree structure propagation in a squared lattice of length $L_{b}$ (m).

The computational procedure can be summarized as follows. First, the solid dielectric geometry and electrode arrangement are modelled using a squared lattice of length $L_{b}$ (m). See Figure 1.

The parameters of media and initial boundary conditions, potential at the HV electrodes, are defined. Second, for each time step, the electric scalar potential, electric field strength, and average energy are calculated by solving the Poisson’s equation using the finite differences and relaxation methods [24]. In addition, Equation (10) is calculated for each bond around the current tree structure. The bond with the highest probability is added to the tree structure by a new channel of length $L_{b}$ (m) and the time step magnitude is calculated from Equation (7). Third, the boundary conditions and vector time are updated and simulations will continue up to the tree structure reaches the plane electrode. The potential in the tree structure is calculated using the relative channel conductivity, $\sigma_{ch}$, approach [25] presented in the Materials and Method section. Although the material for the case of study in this paper is homogeneous, the implemented model is able to consider inhomogeneous materials because the electric potential distribution is calculated solving the Poisson’s equation, Equation (1).

The input parameters of the model are summarized in Table 1.

**Figure 2:**
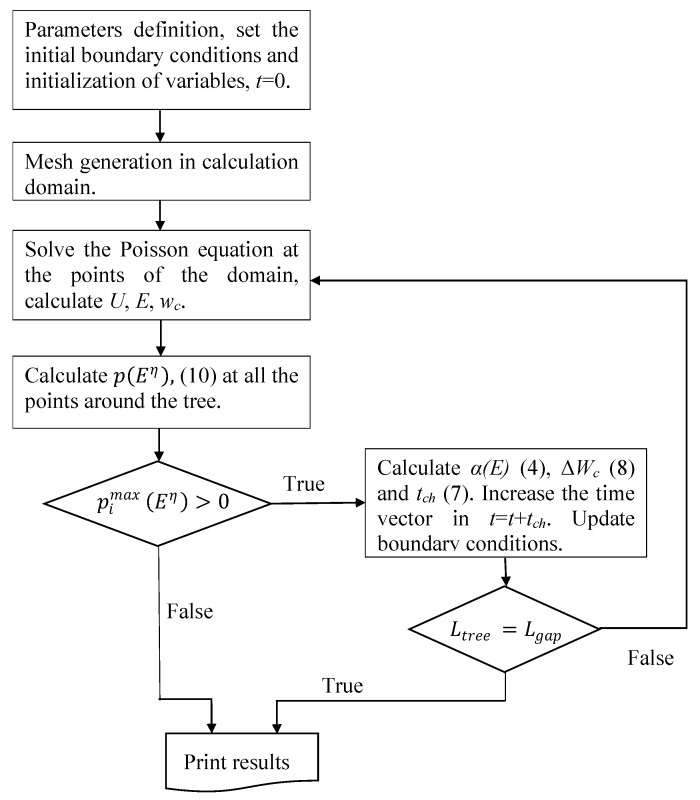
Model implementation flowchart. U (V) is the electric potential, E (V·m^−1^) is the electric field strength magnitude, $w_{c}$ ·(J·m^−3^) is the potential energy density, $L_{tree}$ (m) is the tree length along the symmetry axis and $L_{gap}$ (m) is the pin–plane distance.

In addition, the frequency, *f* (Hz), and the RMS value of the applied voltage, $U_{0}$ (V), are also required. The model was implemented in Matlab and Figure 2 shows the flowchart.

## 5. Results and Discussion

In our simulations, the novel improved model described in the previous section was used. We reproduced the experimental arrangement described in the Materials and Methods section using a 100 × 100 squared lattice, and the pin length was considered as 6 lattice units, which means that $L_{b}$ = 24 μm. According to experimental measurements $N_{c}$ =1 × 10^13^ in tubes 10 μm in length [41], and for this reason $N_{c}$ had to be adjusted proportionally as $N_{c}$ = 1 × 10^13^$L_{b}$/10 μm during simulations in order to consider tubes of different length.

From the experimental cases considered in [22], for comparison purposes, only simulation results for 7, 10 and 15 kV will be presented in this paper. The parameters of the media used for the simulations are presented in Table 2.

It is further assumed that the tree branches are highly conducting, $\sigma_{ch}$ = 1, and the tree structure is equipotential with the pin electrode.

In the original DAM model, a value of $N_{c}/N_{b}$ is assigned to each lattice bond, while a random value from the Gaussian distribution is assigned uniformly to all bonds for each simulation in [21]. Similar to [21], in this study, it is considered that the value of the parameter $N_{c}/N_{b}$ is fixed for each simulation. However, it is considered that its magnitude must be dependent on the applied voltage because the number of avalanches in a half cycle is proportional to the PD rate and this is dependent on the peak value and wave shape of the applied voltage as well as the tree length [57]. The parameters $N_{c}/N_{b}$ and η were adjusted through a trial and error process considering the relative difference between the average value of the time to breakdown obtained from simulations and the measured average breakdown time reported in [22]. A maximum difference of 3% was considered as reasonably tolerable.

**Figure 3:**
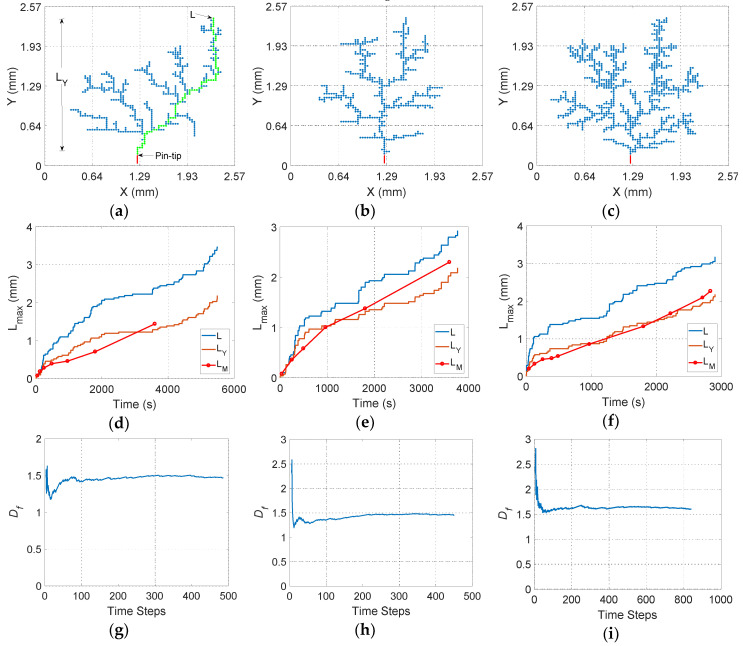
Typical simulation results for the case study. From the top to the bottom: (**a**–**c**) tree structure in 2D; (**d**–**f**) evolution in time of the maximum simulated length (L), the maximum simulated length on the Y axis (L_Y_), and the measured length on the Y axis (L_M_); (**g**–**i**) the calculated fractal dimension. From the left to the right, applied voltage: 7, 10 and 15 kV.

In total, 50 simulations were performed for each voltage in Table 2 and typical simulation results are shown for the three considered voltages in Figure 3. Figure 3 shows the typical simulation results of the tree structure, the characteristic curve of length versus time during tree propagation from the pin tip and the calculated fractal dimension for each time step. From left to right, the simulation results are organized as follows: the simulation results for 7 kV are shown in the first column, Figure 3a,d,g. Results for 10 kV are depicted in the second column, Figure 3b,e,h. Finally, simulations obtained for 15 kV are shown in the third column, Figure 3c,f,i.

In Figure 3d–f, L (m) is the maximum length along the tree structure, the green line in Figure 3a, and L_Y_ (m) is the maximum length of the tree parallel to the symmetry axis, Y, for each time step. See Figure 3a. Additionally, these curves were overlapped with the measured propagation curves, in red lines with solid dots (L_M_), reported as typical in [22]. It should be taken into account that the measured curve is interrupted in Figure 3d since, during the measurement period reported by the authors, 60 min, there was no dielectric breakdown at a voltage of 7 kV.

In Figure 3a–c, it can be seen that the tree structures obtained for the three considered voltages are branched. The highest branch density was obtained for 15 kV, and the lowest for 10 kV. The lower branch density at 10 kV is explained by the greater value of η, which means that the propagation probability is almost proportional to the square of the local electric field strength magnitude. Because of this, the branches are mainly developed in those sites with the highest field magnitudes, that is, along the symmetry axis and parallel to developed main branches. This can be seen in Figure 3b,e where it is shown that the tree initially propagates rapidly along the symmetry axis, Y, where the electric field strength is greater and the time required to create a new channel is low, Equation (7), until it reaches a point, at approximately 1 mm, where the electric field strength magnitude along the radial direction, X axis, is comparable to that on the Y axis. At this time, new branches are created from the branches initially developed close to the pin electrode, where the electric field strength magnitude is greater, and the fractal dimension increases. See Figure 3h.

In Figure 3d–f, it can be seen that for the three considered voltages, the propagation length versus time curves exhibit a similar behavior, that is, there is a rapid propagation initially followed by a deceleration period when the fractal dimension increases—see Figure 3g–i—and finally another acceleration period appears when the tree structure is near to the plane electrode. That behavior can be seen in both the simulated, L_Y_, and measured, L_M_, curves, which allows demonstrating the reliability and efficacy of the model for simulating the physical phenomenon.

In Figure 3e, it can be seen that the initial accelerated growing period is longer at 10 kV than at 7 and 15 kV. This behavior can be seen in the simulated and measured curves. In the model, again, this is because η≅2, which allow us to hypothesize that η could be a parameter of the media related to the rate of energy conversion during the tree propagation. However, more experimentation is required to confirm this hypothesis.

The fractal dimension, *D_f_*, was calculated for each time step using the axial extension method [58]. In Figure 3g–i, it can be seen that the fractal dimension initially increases rapidly, then continues growing at a very low rate, after that, and finally decreases. This final decrement is due to the final accelerated growing, with low branching, when the tree structure is close to the plane electrode. An interesting fact is that the changes in the *D_f_* magnitude are related to the changes in the slope of the propagation curve in Figure 3d–f.

On the other hand, in Figure 3h,i it can be seen that the number of time steps for 15 kV is almost twice that for 10 kV, but the time to breakdown is lower, but not half, for 15 kV than 10 kV, because the energy increases when the applied voltage increases, as described by Equation (8). This allows demonstrating that simplified methods in which the propagation time is considered as proportional to the number of time steps through a conversion constant are not adequate [25,59].

Table 3 shows the simulation results of the fractal dimension calculated for each voltage in Table 2.

The values in the last column of Table 3, Δ*D_f_* (%), correspond to the difference between the average values of the simulated, *D_f-sim_*, and the measured, *D_f-M_*, results with respect to the latter. From the results in Table 3, it is concluded that the model can adequately reproduce the experimentally deducted fractal dimension. The precision of the model, especially for the 7 and 10 kV cases, can be adjusted to reduce the difference (Δ*D_f_*) by modifying the parameter *η* in Table 2. However, it should be noted that the value of the fractal dimension reported in [22] is in fact a calculated value, using the empirical model. In addition, there is no single method to determine this dimension [28].

The distribution of the simulation results for the time to breakdown were adjusted to the Weibull distribution, which is considered adequate for the representation of the probabilities in failure and aging studies [28]. The cumulative probability of failure P for a time *t* (s) is given by:

(11) $$P\left(t\right)=1-\exp\left[-{\left(t/\alpha_{t}\right)}^{\beta }\right],$$

where $\alpha_{t}$ is the characteristic time parameter and β is the shape parameter. These parameters are estimated using the maximum likelihood method [60]. The calculated values of $\alpha_{t}$ and β as well as the limits of the 95% confidence intervals, represented with the angled brackets, are shown in Table 4 for the time to breakdown distribution.

The Δ*t_m_* (%) values in the last column of Table 4 correspond to the difference between the average time to breakdown obtained in the simulations, *t_mean_* (s), and the average value reported from the measurements, *t_meas_* (s), with respect to the latter. The maximum difference was obtained for 7 kV, which can be reduced changing the value of the $N_{c}/N_{b}$ parameter in Table 2. However, it is considered that 2.4% is a reasonably tolerable difference. An ideal comparison also requires the analysis of $\alpha_{t}$. However, the paper from which this case study was taken does not include this value for the experimental results.

In terms of the voltages analyzed, it can be concluded that both the time to breakdown and the 63% probability quantile, $\alpha_{t}$, decrease almost linearly when the voltage increases. This conclusion is totally confirmed based on simulation results because there is no overlapping in the confidence intervals of $\alpha_{t}$.

From the results presented in Figure 3, Table 3 and Table 4, and on comparing these with the experimentally measured values in [22], it is concluded that the model allows obtaining values of time to breakdown and fractal dimension that are within the ranges of the measured values. See Table 4. For the time to breakdown, as in the experimental cases, there is high dispersion, which is explained by the values of the parameters used in the simulation, since values $N_{c}/N_{b}$, I and λ are reported as typical for polymers based on measurements made of polyester [21]. Results that are more accurate can be achieved by modifying those parameters. However, said modification should be the result of measurements of those parameters in the type of resins used for the simulations. On the other hand, from our experience in PD simulation at different defects [61] and measurements reported by other authors [56], it is considered that the parameter $N_{c}/N_{b}$ must be variable along the tree propagation because it is dependent on the PD behavior, which is variable, in magnitude and rate, during the tree propagation. A further experimental study should be focused on the evolution of the $N_{c}/N_{b}$ parameter as the tree propagates.

The implemented model yields a propagation length versus time curve quite similar to that found experimentally, which describes a shape similar to an inverted sigmoidal, with an accelerated growth at the beginning, followed by a deceleration period and an uncontrolled accelerated growth at the end until the breakdown. This can be seen by comparing the curves—orange, simulated, and red with solid dots—measured experimentally, in Figure 3d–f. Both curves not only have a similar shape but also have a similar rate during propagation, which allows us to demonstrate the validity of the proposed physical model. It should be pointed out here that most of the other existing physical models, except for the empirical ones, are unable to reproduce the changes in the propagation characteristic curve over time measured experimentally, whereas the model presented here can. So this model can be considered as an improvement to the physical models. On the other hand, other stochastic models do not directly involve time as a physical variable. However, the proposed model does this through energy analysis, so this model can be considered as an improvement to stochastic-physical models.

From results in Table 3 and Table 4, it is concluded that there is no direct linear correlation between the fractal dimension and the time to breakdown, as deducted from empirical methods [22], and the applied voltage must be considered in that relationship because the time to breakdown is dependent on the applied voltage through the potential energy.

The parameters $N_{c}/N_{b}$, I, η and $E_{c}$ allow simulating tree propagation in materials under different conditions—for example, with variations in the glass transition temperature, flexible and rigid materials can be taken into account through $E_{c}$ and the conductivity of media [62].

On the other hand, non-homogeneity conditions can be considered, taking into account the conductivity of the channels of the tree structure, composite materials, NPs, and space charge, among others.

## 6. Conclusions

A review of the different models for simulating tree propagation in solid dielectric was carried out and a new improved physical-stochastic model, based on the DAM model, for tree propagation in polymeric materials was proposed using an energy analysis approach. The novel improved model allows quantitatively and qualitatively analyzing the electrical tree propagation behavior under different material conditions and applied stresses. In addition, the model is able to adequately predict the time to breakdown and propagation curves, which describes the same dynamical changes during tree propagation as found in experimental studies.

Furthermore, parameters of the media required for implementing the simulations can be determined experimentally or typical values reported in the literature can be used. The physical relationship between the parameters and the propagation process was discussed. In addition, a revision of the critical number of avalanches per half cycle as a constant parameter was proposed, taking into account its dependency on the PD behavior as the tree propagates. The novel model is implemented using a 2D squared lattice, and for this reason, only electrical trees with a fractal dimension below two can be successfully simulated. However, the model can be extended to a 3D lattice using the same approach if a 3D solver of the Equation (1) is employed.

On the other hand, from the simulation results, it was shown that the time to breakdown reduces when the applied voltage increases because there is no overlapping in the confidence intervals. In addition, it was found that there is no direct correlation between the fractal dimension and the time to breakdown, and further there is no direct correlation between the fractal dimension and applied voltage. There is an inverse relationship between the fractal dimension and the magnitude of the parameter η—*D_f_* increases when η decreases and vice versa. It was hypothesized that η could be a parameter of media related to the rate of energy conversion during the tree propagation. However, this requires experimental confirmation.

Finally, other simulations, considering different conditions of materials, space charge, frequency and applied voltage, could be implemented using the novel proposed model and their simulation results will be implemented and their results presented in another document.

## Figures and Tables

**Table 1:** Input parameters for the proposed improved model.

Parameter	Description
λ(m)	Mean free path, depends on the material and experimental conditions, for polymers 60–120 nm [21]
I(eV)	Ionization energy, depends on the material, for polymers 8–10 eV [53]
$\sigma_{ch}$	Relative conductivity of the tree channels—0 for non-conductive channels and 1 for highly conductive channels [25]. Depends on the material and experimental conditions [56]
$\varepsilon_{r}$	Relative permittivity of media, depends on the materials. It is assumed that the tree channel and solid dielectric have the same permittivity
$N_{c}/N_{b}$	Critical number of avalanches per half cycle and electron is considered a random magnitude from the Gaussian distribution truncated at the maximum value of *N_c_*/*N_b_* = 1 × 10^8^ [33]
$E_{c}$(V·m^−1^)	Critical magnitude of electric field strength for tree propagation, depends on the material [37]. Also can be calculated from Equation (9)
η	Power for stochastic model (0–2), depends on the experimental conditions [27,29,30]

**Table 2:** Parameters of media used for simulating the case study, taken from [21,22].

Voltage (kV RMS)	*I* (eV)	*λ* (nm)	*N_c_/N_b_*	*Ec* (kV·mm^−1^)	*ε_r_*	*η*
7	8	60	1 × 10^7^	14	4	1
10	8	60	4 × 10^8^	14	4	1.8
15	8	60	4 × 10^8^	14	4	1

**Table 3:** Fractal dimension results. *D_f-sim_*, simulated; *D_f-M_*, measured. Measured values from [22].

Voltage (kV RMS)	*D_f-sim_*	*D_f-M_*	Δ*D_f_* (%)
7	1.55	1.65	6.06
10	1.50	1.55	3.23
15	1.64	1.66	1.21

**Table 4:** Weibull distribution parameters for the time to breakdown. Typical measured breakdown time *t_meas_* taken from [22].

Voltage (kV RMS)	*β*	*α_t_* (s)	*t_min_* (s)	*t_max_* (s)	*t_mean_* (s)	*t_meas_* (s)	Δ*t_m_* (%)
7	8.3 < 10.4 < 12.9	5576.9 < 5735.4 < 5898.5	4211.2	6589.6	5457.6	5329.4	2.4
10	4.5 < 5.6 < 6.9	3457.2 < 3644.5 < 3842.0	2262.1	4881.1	3370.3	3404.3	0.9
15	6.6 < 8.1 < 9.9	2813.0 < 2916.9 < 3024.6	2074.0	3577.2	2753.7	2812.1	2.1
